# HIV-1 drug resistance before initiation or re-initiation of first-line antiretroviral therapy in low-income and middle-income countries: a systematic review and meta-regression analysis

**DOI:** 10.1016/S1473-3099(17)30702-8

**Published:** 2018-03

**Authors:** Ravindra K Gupta, John Gregson, Neil Parkin, Hiwot Haile-Selassie, Amilcar Tanuri, Liliana Andrade Forero, Pontiano Kaleebu, Christine Watera, Avelin Aghokeng, Nicholus Mutenda, Janet Dzangare, San Hone, Zaw Zaw Hang, Judith Garcia, Zully Garcia, Paola Marchorro, Enrique Beteta, Amalia Giron, Raph Hamers, Seth Inzaule, Lisa M Frenkel, Michael H Chung, Tulio de Oliveira, Deenan Pillay, Kogie Naidoo, Ayesha Kharsany, Ruthiran Kugathasan, Teresa Cutino, Gillian Hunt, Santiago Avila Rios, Meg Doherty, Michael R Jordan, Silvia Bertagnolio

**Affiliations:** a Department of Infection, University College London, London, UK; b Africa Health Research Institute, Durban, South Africa; c Department of Statistics, London School of Hygiene & Tropical Medicine, London, UK; d Data First Consulting, Belmont, CA, USA; e HIV Department, World Health Organization, Geneva, Switzerland; f Federal University of Rio de Janeiro, Rio de Janeiro, Brazil; g Ministry of Health, Bogotá, Columbia; h Uganda Virus Research, Entebbe, Uganda; i Medical Research Council and Uganda Virus Research Institute, Uganda Research Unit, Entebbe, Uganda; j CREMER, Virology laboratory IMPM-IRD, IMPM, Yaoundé, Cameroon; k IRD UMI 233, INSERM U1175, Université de Montpellier, Montpellier, France; l Ministry of Health, Windhoek, Namibia; m Ministry of Health and Child Care, Harare, Zimbabwe; n Department of Public Health, Ministry of Health and Sports, Yangoon, Myanmar; o Department of Epidemiology, Guatemala City, Guatemala; p HIV Program, Ministry of Health, Guatemala City, Guatemala; q HIV Reference Laboratory, Guatemala City, Guatemala; r Ministry of Health, Managua, Nicaragua; s Universidad del Valle de Guatemala Centro de Estudios en Salud, Guatemala City, Guatemala; t Amsterdam Institute for Global Health and Development, Amsterdam, Netherlands; u Department of Global Health, Academic Medical Centre of the University of Amsterdam, Amsterdam, Netherlands; v Laboratory Medicine, Global Health and Medicine, University of Washington, Seattle, WA, USA; w Seattle Children's Research Institute, Seattle, WA, USA; x Departments of Global Health, Medicine, and Epidemiology, University of Washington, Seattle, WA, USA; y KwaZulu-Natal Research Innovation and Sequencing Platform (KRISP), Nelson R Mandela School of Medicine, University of KwaZulu-Natal, Durban, South Africa; z Centre for the AIDS Programme of Research in South Africa (CAPRISA), Durban, South Africa; aa MRC-CAPRISA HIV-TB Pathogenesis and Treatment Research Unit, Doris Duke Medical Research Institute, University of KwaZulu-Natal, Durban, South Africa; ab Department of Infection, University College London Hospital, London, UK; ac National Institute for Communicable Diseases, Sandringham, South Africa; ad Centre for Research in Infectious Diseases, National Institute of Respiratory Diseases, Mexico City, Mexico; ae Department of Public Health and Community Medicine, Tufts University School of Medicine, Boston, MA, USA; af Division of Geographic Medicine and Infectious Disease, Tufts Medical Center, Boston, MA, USA

## Abstract

**Background:**

Pretreatment drug resistance in people initiating or re-initiating antiretroviral therapy (ART) containing non-nucleoside reverse transcriptase inhibitors (NNRTIs) might compromise HIV control in low-income and middle-income countries (LMICs). We aimed to assess the scale of this problem and whether it is associated with the intiation or re-initiation of ART in people who have had previous exposure to antiretroviral drugs.

**Methods:**

This study was a systematic review and meta-regression analysis. We assessed regional prevalence of pretreatment drug resistance and risk of pretreatment drug resistance in people initiating ART who reported previous ART exposure. We systematically screened publications and unpublished datasets for pretreatment drug-resistance data in individuals in LMICs initiating or re-initiating first-line ART from LMICs. We searched for studies in PubMed and Embase and conference abstracts and presentations from the Conference on Retroviruses and Opportunistic Infections, the International AIDS Society Conference, and the International Drug Resistance Workshop for the period Jan 1, 2001, to Dec 31, 2016. To assess the prevalence of drug resistance within a specified region at any specific timepoint, we extracted study level data and pooled prevalence estimates within the region using an empty logistic regression model with a random effect at the study level. We used random effects meta-regression to relate sampling year to prevalence of pretreatment drug resistance within geographical regions.

**Findings:**

We identified 358 datasets that contributed data to our analyses, representing 56 044 adults in 63 countries. Prevalence estimates of pretreatment NNRTI resistance in 2016 were 11·0% (7·5–15·9) in southern Africa, 10·1% (5·1–19·4) in eastern Africa, 7·2% (2·9–16·5) in western and central Africa, and 9·4% (6·6–13·2) in Latin America and the Caribbean. There were substantial increases in pretreatment NNRTI resistance per year in all regions. The yearly increases in the odds of pretreatment drug resistance were 23% (95% CI 16–29) in southern Africa, 17% (5–30) in eastern Africa, 17% (6–29) in western and central Africa, 11% (5–18) in Latin America and the Caribbean, and 11% (2–20) in Asia. Estimated increases in the absolute prevalence of pretreatment drug resistance between 2015 and 2016 ranged from 0·3% in Asia to 1·8% in southern Africa.

**Interpretation:**

Pretreatment drug resistance is increasing at substantial rate in LMICs, especially in sub-Saharan Africa. In 2016, the prevalence of pretreatment NNRTI resistance was near WHO's 10% threshold for changing first-line ART in southern and eastern Africa and Latin America, underscoring the need for routine national HIV drug-resistance surveillance and review of national policies for first-line ART regimen composition.

**Funding:**

Bill & Melinda Gates Foundation and World Health Organization.

## Introduction

The scale-up of antiretroviral therapy (ART) for the treatment of HIV has reached 19·5 million individuals globally and is an unprecedented public health achievement.^1^ Despite this accomplishment, millions more people with HIV need to initiate and be maintained on ART for life. WHO and UNAIDS have set ambitious targets to end the AIDS epidemic as a public health threat by 2030. These widely adopted targets reflect the global community's commitment to expanding access to ART and are aiming, by 2020, to diagnose 90% of all people with HIV infection, provide treatment to 90% of those diagnosed, and ensure that 90% of people on treatment achieve virological suppression.^2^ As ART scale-up proceeds, some degree of HIV drug resistance is anticipated and will have to be managed. Should the prevalence of HIV drug resistance in people starting treatment rise to substantial levels, global efforts to achieve the so-called third 90 might be in danger, thereby warranting policy and guideline changes.

**Research in context**

**Evidence before this study**

We searched PubMed for meta-analyses of pretreatment HIV-1 drug resistance over time in adults starting antiretroviral therapy (ART) in low-income and middle-income countries (LMICs), published in English, Spanish, or Portuguese. We limited our search to studies published between Jan 1, 2012, and Aug 31, 2017, because we were interested in contemporary trends and prevalence estimates for drug resistance. We used the search terms “HIV” AND “transmitted HIV drug resistance” AND “systematic review”; “HIV” AND “pretreatment drug resistance” AND “systematic review”; “HIV” AND “transmitted drug resistance” AND “meta-analysis”; “HIV” AND “pretreatment drug resistance” AND “meta-analysis”. We did not identify any such studies in adults.

**Added value of this study**

Our findings provide up-to-date estimates of the prevalence of HIV drug resistance in people initiating or re-initiating first-line ART and we found worrying increases in prevalence in all regions of sub-Saharan Africa, Asia, and Latin America and the Caribbean. The prevalence of HIV drug resistance seems to be 10% or higher in several regions and was much higher in studies in which individuals reported previous antiretroviral exposure. We also noted an increase in virological failure after first-line ART in individuals who reported previous antiviral exposure.

**Implications of all the available evidence**

Our results show that some LMICs might be reaching WHO's 10% threshold for changing first-line non-nucleoside reverse transcriptase inhibitor (NNRTI)-based ART to integrase inhibitor-based ART. Individuals with previous ART exposure should be identified and NNRTI-based regimens should be avoided in this group.

In 2010, WHO reported that prevalence estimates for HIV resistance to the non-nucleoside reverse transcriptase inhibitor (NNRTI) backbone of first-line ART reached 5·5% in low-income and middle-income countries (LMICs).^3^ Currently, global HIV treatment guidelines recommend first-line ART based on the NNRTI efavirenz in combination with two nucleoside reverse transcriptase inhibitors (NRTIs), usually tenofovir and either lamivudine or emtricitabine.^4^ A 2015 systematic review and meta-analysis reported that 20% of adults receiving ART for 12–60 months in LMICs had unsuppressed viral loads.^5^ Among people with treatment failure on ART, between 70% and 90% have drug-resistant virus, with most resistance to the NNRTI drug class.^6^, ^7^, ^8^ Transmission of drug-resistant virus to newly infected people has been identified as a key challenge^9^ and is particularly relevant given the increased risk of treatment failure in people who have NNRTI resistance and who start an NNRTI-based ART regimen.^10^, ^11^, ^12^

With ART becoming increasingly available in LMICs, an increasing number of people initiating NNRTI-containing ART are not actually antiretroviral-naive but instead have disclosed or undisclosed previous exposure to antiretroviral drugs resulting from treatment for the prevention of mother-to-child transmission of HIV or previous disengagement from care.^13^ In nationally representative surveys of HIV drug resistance, the proportion of people self-reporting previous exposure to antiretroviral drugs at the time of re-initiating NNRTI-based ART was high.^13^ In a large study from South Africa, 24% of the 326 first-line ART initiators reported previous exposure to antiretroviral drugs.^14^ Previous use of antiretroviral drugs before ART initiation is becoming increasingly recognised as problematic in LMICs because individuals with previous antiretroviral drug exposure are at high risk of having HIV drug resistance^7^, ^15^ and when initiating or re-initiating an NNRTI-containing first-line ART regimen, might be more at risk of treatment failure.

The 2016 WHO consolidated guidelines on the use of antiretroviral drugs for the treatment and prevention of HIV infection recommend an NNRTI-based regimen for all populations starting or restarting ART, regardless of previous use of antiretroviral drugs, except in children younger than 3 years.^4^ Most LMICs do not differentiate between people initiating or re-initiating first-line ART and thus do not take the risk of resistance due to previous exposure into consideration when making treatment recommendations as the same NNRTI-based regimen is offered to both initiators and re-initiators. Although previous exposure to antiretroviral drugs is likely to be associated with HIV drug resistance, no systematic assessment of the risk of resistance and treatment outcomes in this population has been done.

Unchecked emergence of HIV drug resistance could have real world consequences: modelling suggests that, where the population prevalence of HIV drug resistance in people initiating or re-initiating ART (pretreatment drug resistance) exceeds 10%, resistant virus could result in 890 000 deaths due to AIDS and 450000 new infections in sub-Saharan Africa alone during the period 2016–30 if no action is taken and NNRTIs continue to be used in first-line ART.^16^ To counter this threat, in July, 2017, WHO released guidelines on the public health response to pretreatment drug resistance for countries reporting high (>10%) prevalence of pretreatment resistance to NNRTIs among people starting or restarting first-line ART.^17^ Assessment of recent levels and trends of pretreatment drug resistance in LMICs is therefore crucial in the global response to HIV/AIDS. We did this study to help fill this knowledge gap.

## Methods

### Search strategy and selection criteria

This study was a systematic review and meta-regression analysis of the regional prevalence of pretreatment drug resistance and risk of pretreatment istance among ART initiators reporting previous ART exposure. We searched for studies in PubMed and Embase and conference abstracts and presentations from the Conference on Retroviruses and Opportunistic Infections, the International AIDS Society Conference, and the International Drug Resistance Workshop for the period Jan 1, 2001, to Dec 31, 2016. We supplemented the systematic review with additional unpublished datasets from WHO-supported surveys of drug resistance.

We searched for studies in adults (aged >15 years) infected with HIV who were eligible to initiate first-line NNRTI-based ART in LMICs in the WHO-defined regions western Pacific, southeast Asia, Africa, eastern Mediterranean, and Latin America and the Caribbean. For the purpose of this analysis, countries in the southeast Asia, western Pacific region, eastern Mediterranean regions, as well as Turkey (Europe region) are grouped under the regional heading of “Asia”. We analysed data within subregions of sub-Saharan Africa: eastern Africa, southern Africa, and west and central Africa. Because we included studies from only resource-limited settings, we used the search term “Latin America and the Caribbean” instead of the “Americas”. We excluded studies with fewer than ten HIV-1 genotypes. We used the search terms “antiretroviral therapy” AND “transmitted drug resistance”; “antiretroviral therapy” AND “pretreatment drug resistance”; “antiretroviral therapy” AND “(stavudine OR zidovudine OR nevirapine OR efavirenz)”; “HIV” AND “transmitted drug resistance”; “antiretroviral therapy” AND “(stavudine OR zidovudine OR nevirapine OR efavirenz)”; “HIV” AND “pretreatment drug resistance”; “HIV” AND “antenatal; “HIV” AND “VCT”; “genotyp*” AND “HIV” AND “naive”; “genotyp*” AND “HIV” AND “resistance”; and “genotyp*” AND “HIV” AND “resistance” AND “primary”, with the search restricted to records in English, Spanish, or Portuguese. We did not contact study authors for unavailable data.

The following study-level data were extracted from each study: country, year of sample collection, sex, risk groups, setting, pretreatment CD4 cell count, number of pretreatment genotypes reported in the study, and exposure to antiretroviral drugs prior to treatment initiation (yes, no, or unknown). Additionally, the number of people with more than one drug-resistance mutation, one or more NRTI mutations, one or more thymidine analogue mutations, one or more NNRTI mutations, and one or more protease inhibitor mutations were extracted. When individual sequences were made available for analysis, drug resistance-mutations were defined as those appearing on the 2009 WHO surveillance drug resistance-mutations list.^18^ In all other cases, study authors' interpretations of HIV drug resistance based on the Stanford HIVdb algorithm, International Antiviral Society-USA mutations list, and the Agence autonome de l'Inserm algorithms mutations list were used. RKG JGr RK and TC did the searches and data extraction. Conflicts over inclusion were decided by RKG.

### Data analysis

The two major aims of our study were to characterise changes in drug resistance over time and to compare the prevalence of drug resistance in people restarting ART after reported previous antiretroviral exposure versus treatment-naive patients. We also assessed whether factors including sex, CD4 cell count, rural or urban setting, or risk groups were predictors of HIV drug resistance in any geographical region. We therefore extracted information on drug resistance separately for each calendar year, treating each year as a separate datapoint in our analyses. We extracted information separately for patients before treatment initiation with and without prior exposure (reported or unreported) to antiretroviral drugs in studies where this information was available. The database was manually scanned for duplicates by JGr, NP and RKG. Where there were duplicate publications, the publication with information on the largest number of genotypes was used.

Statistical analysis was done in Stata version 14.1. To assess the prevalence of drug resistance within a specified region at any specific timepoint, we pooled prevalence estimates within the region using an empty logistic regression model with a random effect at the study level (appendix pp 20, 21). We did not formally assess study quality.

To assess associations between study-level characteristics (eg, calendar year at the midpoint of the study year) and drug resistance, we used univariate meta-regression analyses within each region (appendix pp 20, 21). We also explored the prevalence of specific drug-resistance mutations among all individuals with any WHO surveillance drug-resistance mutations. We calculated the proportion with specific mutations after crudely pooling the numbers of individuals with any mutation and the number with specific mutations. Heterogeneity was assessed using the *I*^2^ statistic.

### Role of the funding source

The funders of the study had no role in study design, data collection, data analysis, data interpretation, or writing of the report. RKG had full access to all the data in the study and had final responsibility for the decision to submit for publication.

## Results

We initially identified 19 458 potential studies or reports of 697 full-length papers assessed. 339 records were excluded on the basis of our eligibility criteria. We included 358 datasets in our analysis, which represented 56 044 adults with data on HIV drug resistance across 63 countries (appendix pp 1–13). 277 (93%) of 299 dataset with unambiguous location information were derived from urban settings (table 1). The identified studies included 23 948 genotypes from sub-Saharan Africa, (42·7% of all genotypes), 16 008 (28·6%) from Latin America and the Caribbean, and 16 088 (28·7%) from Asia. The median number of genotypes per study was 95 (table 1). There was a large amount of variation in the prevalence of drug-resistant mutations reported by studies, even within geographical regions (appendix p 14). We did not find study level summaries of sex or CD4 cell count to be associated with the prevalence of HIV drug resistance in any region (appendix pp 15–17). We detected an association between men who have sex with men and overall HIV drug resistance in Asia (p=0·047), but not for NNRTI or NRTI resistance specifically or in other geographical regions. We found insufficient data to assess other risk groups or the impact of rural versus urban or peri-urban setting on HIV drug resistance.

**Table 1:** Characteristics of included studies by region

	**Number of studies**	**Number of genotypes**	**Genotypes per study**	**Sampling year**	Studies in urban populations*
Eastern Africa	53	7169	92 (57–187)	2008 (2005–09)	32/44 (73%)
Southern Africa	61	11 855	102 (53–108)	2007 (2004–09)	41/47 (87%)
Western and central Africa	56	4924	79 (49–104)	2007 (2004–09)	48/50 (96%)
Latin America and the Caribbean	90	16 008	98 (52–221)	2008 (2003–10)	67/69 (97%)
Asia†	98	16 088	97 (47–223)	2009 (2006–10)	89/89 (100%)
Overall	358	56 044	95 (50–194)	2008 (2005–10)	277/299† (93%)

Data are n, median (IQR), or n/N (%).

* Denominators restricted to studies with unambiguous information on location available.

† For the purpose of this analysis, countries in the southeast Asia, the western Pacific, and eastern Mediterranean regions and Turkey (Europe region) are grouped under the regional heading of Asia.

**Figure 1:**
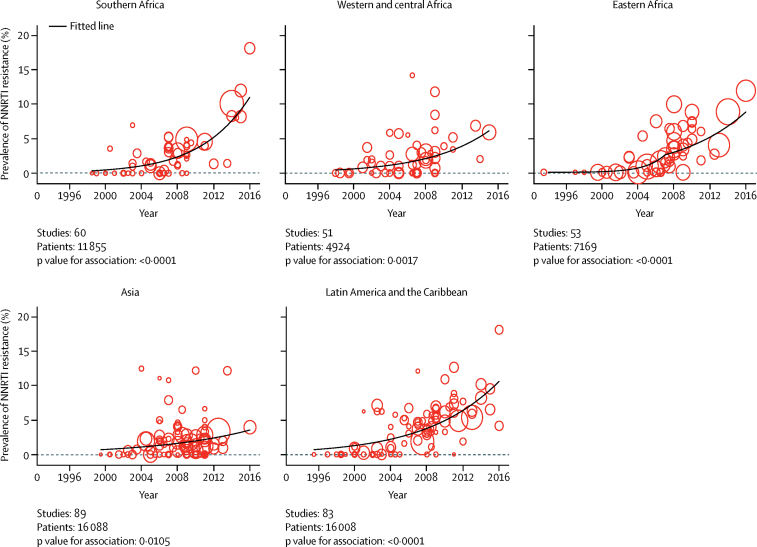
Prevalence of pretreatment HIV resistance to NNRTI inhibitors by year of sampling Each bubble represents a study and the size of the bubble is proportional to the size of the study. NNRTI=non-nucleoside reverse transcriptase.

NNRTI resistance was more prevalent in more recent studies than in older studies across all regions (p<0·05 for all regions; figure 1; appendix p 18). Annual increases in the odds of pretreatment NNRTI resistance per year were 23% (95% CI 16–29) in southern Africa, 17% (6–29) in western and central Africa, and 11% (2–20) in Asia. In eastern Africa and Latin America and the Caribbean, there was evidence that the annual increase in the odds of pretreatment drug resistance, but not necessarily absolute changes in pretreatment drug resistance, have declined in more recent years (p for change in odds over time 0·027 for eastern Africa and 0·033 for Latin America and the Caribbean). Estimated annual increases in the odds of pretreatment NNRTI resistance since 2007 were 17% (5–30) in eastern Africa and 11% (5–18) in Latin America and the Caribbean. The model-predicted year-on-year increases in prevalence levels of NNRTI resistance between 2015 and 2016 were 1·8% in southern Africa, 1·3% in eastern Africa, 1·0% in western and central Africa, 0·9% in Latin America and the Caribbean, and 0·3% in Asia. Modelled prevalence estimates of pretreatment NNRTI resistance in 2016 were 11·0% (7·5–15·9) in southern Africa, 10·1% (5·1–19·4) in eastern Africa, 7·2% (2·9–16·5) in western and central Africa, 9·4% (6·6–13·2) in Latin America and the Caribbean, and 3·2% (1·8–5·6) in Asia (figure 1). As expected, the prevalence of NNRTI resistance in studies done between 2014 and 2016 agreed well with model-predicted prevalence levels for 2016 in all regions (appendix p 18).

**Figure 2:**
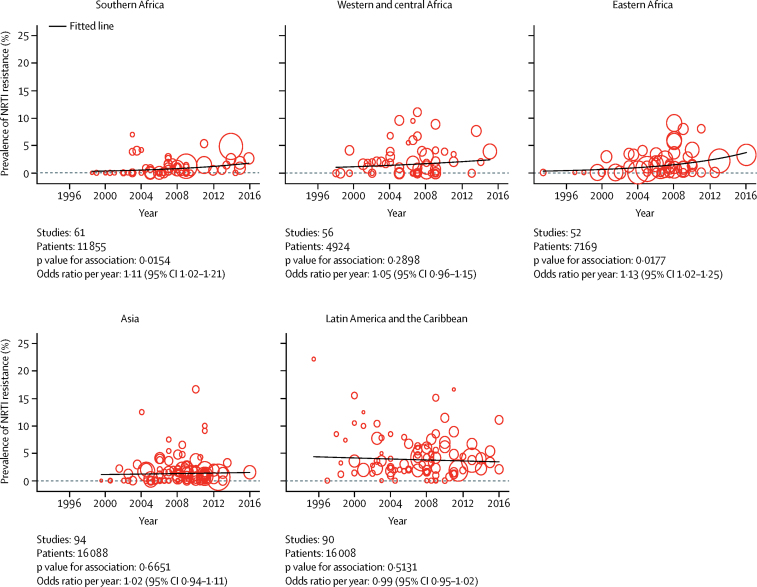
Prevalence of pretreatment HIV resistance to NRTI by year of sampling Each bubble represents a study and the size of the bubble is proportional to the size of the study. NRTI=nucleoside reverse transcriptase inhibitors.

By contrast with the increasing prevalence of NNRTI resistance over time, we saw little change in the prevalence of NRTI resistance, with model predicted prevalence in 2016 below 5% in all regions (figure 2). For Latin America and the Caribbean, western and central Africa, and Asia, there was no discernible trend in NRTI resistance over time (p>0·05 for all), and although increases in NRTI resistance were significant over time in southern Africa (p=0·015) and eastern Africa (p=0·017), changes in the prevalence of resistance were small (figure 2).

**Figure 3:**
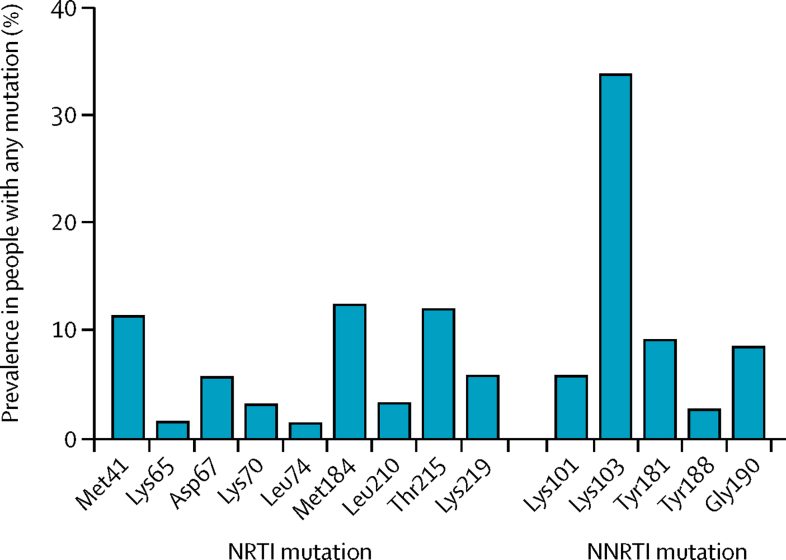
Crude prevalence of reverse transcriptase drug-resistance mutations in people with any mutation NRTI=nucleoside reverse transcriptase inhibitors. NNRTI=non-nucleoside reverse transcriptase inhibitor.

The most common NNRTI mutations (present in 10% or more of individuals with pretreatment drug resistance) from the WHO surveillance drug-resistance mutations list were Lys103Asn (present in 793 [34%] of 2349 people with pretreatment drug resistance), Tyr181Cys (215 [9%] patients), and Gly190Ala (200 [9%] patients; figure 3). The most common NRTI mutation was Met184Ile/Val (292 [12%] patients). Tenofovir resistance (Lys65Arg/Asn or Leu74Val/Ile) was relatively uncommon (3% of patients; patient-level data not available), although the thymidine analogue mutations Asp67Asn (134 [6%] patients) and Met41Leu (267 [11%] patients), which confer resistance to zidovudine, were more common. The prevalence of drug resistance to protease inhibitors was universally very low (<1%). The total number of drug-resistance mutations was available from 249 studies involving 29 898 patients in total and 1452 patients with any drug-resistant mutation. In these 1452 patients, the mean number of mutations per patient was 1·53 (SD not available from study-level data).

In a subset of 27 studies with 6534 patients, information was available on the presence of previous antiretroviral exposure. Previous antiretroviral exposure occurred in 538 (8%) patients and was associated with substantially higher NNRTI resistance. Despite scarce data for many geographical regions, we were able to detect significant differences in the prevalence of pretreatment NNRTI resistance between patients with and without previous antiretroviral drug exposure in Asia, Latin America and the Caribbean, and southern Africa (table 2). Differences were not significant in eastern Africa, or western and central Africa; although statistical power was limited, particularly for western and central Africa where few patients had prior exposure (table 2; appendix p 19). NRTI resistance was also more common among individuals with previous antiretroviral drug exposure compared with antiretroviral drug naive in all regions, with significant differences in all regions except for east Africa (table 2; appendix p 19).

**Table 2:** Prevalence of HIV-1 drug resistance among antiretroviral-naive individuals compared with those starting first-line ART reporting prior antiretroviral drug exposure

	**Studies with data on previous treatment**	**Treatment naive**		**Previous treatment**		Odds ratio (95% CI)*	**p value**
		Number of patients	Proportion (95% CI)	Number of patients	Proportion (95% CI)		
**Any resistance**							
Asia	4	1030	3·8% (2·8–5·1)	73	24·0% (7·0–56·9)	6·35 (2·15–18·76)	<0·0001
Eastern Africa	7	1790	8·7% (6·4–11·8)	91	17·6% (11·1–26·8)	2·31 (1·36–3·92)	0·023
Latin America	4	797	11·7% (9·6–14·1)	138	43·6% (21·6–68·5)	3·46 (2·07–5·81)	<0·0001
Southern Africa	9	1810	4·9% (3·2–7·3)	203	34·3% (27·5–41·9)	7·42 (3·86–14·26)	<0·0001
Western and central Africa	2	328	2·4% (0·9–4·4)†	4	25·0% (3·4–76·2)	15·68 (not estimable)	0·085
**NNRTI resistance**							
Asia	4	1030	2·6% (1·6–4·0)	73	19·8% (7·6–42·8)	8·05 (4·25–15·26)	<0·0001
Eastern Africa	7	1790	6·0% (3·9–9·1)	91	9·9% (5·2–17·9)	2·04 (1·07–3·89)	0·30
Latin America	4	797	9·2% (7·3–11·4)	138	32·8% (15·1–57·2)	3·40 (1·79–6·48)	<0·0001
Southern Africa	9	1810	3·8% (2·3–6·0)	203	31·5% (23·3–40·9)	8·31 (4·23–16·32)	<0·0001
Western and central Africa	3	569	2·9% (1·5–5·8)	33	12·1% (4·6–28·2)	6·89 (0·41–117·23)	0·061
**NRTI resistance**							
Asia	4	1030	1·6% (1·0–2·5)	73	9·9% (0·6–65·7)	13·29 (2·29–77·03)	0·00034
Eastern Africa	6	1790	3·5% (1·8–6·7)	58	6·9% (2·6–17·0)	2·76 (0·56–13·53)	0·37
Latin America	4	797	3·4% (1·9–6·0)	138	15·5% (6·9–31·4)	4·58 (1·96–10·71)	<0·0001
Southern Africa	9	1810	0·7% (0·4–1·4)	203	7·9% (4·9–12·5)	9·53 (4·17–21·76)	<0·0001
Western and central Africa	4	569	0·7% (0·3–1·9)	81	6·2% (0·3–61·5)	40·89 (not estimable)	<0·0001

p values are for the difference between treatment-naive and previously treated people using random effects meta-regression. Where feasible, drug-resistance mutations were defined as those appearing on the 2009 WHO surveillance drug-resistance mutations list. Otherwise, the study authors' interpretation was used.

* Odds ratios use a random effects meta-analyses of within-study odds ratios.

† Uses Freeman-Tukey arcsin transformation because mixed models did not converge. NRTI=nucleoside reverse-transcriptase inhibitor. NNRTI=non-nucleoside reverse-transcriptase inhibitor.

We identified a subset of 13 studies with prospective data on treatment outcomes in adults in Africa who reported previous antiretroviral drug exposure. People who initiated NNRTI-based ART and self-reported previous antiretroviral drug exposure (n=83) had significantly greater risk of virological failure (defined as a viral load of 1000 copies per mL) at 12 months than antiretroviral-naive people (n=1944; odds ratio 2·91, 95% CI 1·48–5·72 after adjustment for age, baseline viral load, baseline CD4 cell count, initial ART, adherence, WHO clinical stage, year of ART initiation, sex, and pretreatment drug resistance, p=0·002).

## Discussion

Pretreatment HIV drug resistance can be detected in people naive to antiretroviral drugs who are initiating ART or people who are initiating or re-initiating first-line ART who have had previous exposure. Pretreatment drug resistance can be either transmitted or acquired drug resistance, or both. This resistance could have been transmitted at the time of infection (ie, transmitted drug resistance), or it might be acquired after antiretroviral drug exposure: eg, in women exposed to antiretroviral drugs for the prevention of mother-to-child transmission of HIV, people who have received pre-exposure prophylaxis, people re-initiating first-line ART after a period of treatment interruption without documented virological failure, or off-prescription use of ART through sharing within families or friends or black market availability.

Our analysis shows that the prevalence of pretreatment drug resistance is rising in many LMICs. This situation contrasts with that in high-income regions where the prevalence of pretreatment drug resistance has been in decline over the past decade and has stabilised at around 10%.^19^ WHO 2016 antiretroviral guidelines recommend the use of NNRTI as a component of first-line ART regimens,^4^ which is widely implemented in LMICs, irrespective of previous exposure to antiretroviral drugs. A single aminoacid mutation can confer resistance to efavirenz or nevirapine, and the presence of NNRTI resistance is particularly crucial to predict the efficacy of recommended first-line regimens.^10^ Our analysis shows that, in several regions, pretreatment NNRTI resistance in populations initiating ART has reached levels exceeding the recently established 10% prevalence threshold above which countries should urgently consider responding. This response could be by either introducing a non-NNRTI-based first-line regimen or, in circumstances where use of a non-NNRTI-containing first-line regimen is not feasible because of cost or other considerations, using pretreatment drug-resistance testing to guide first-line ART regimen selection, if laboratory infrastructure and costs allow.^17^

To our knowledge, our analysis provides the first robust evidence that people with disclosed previous antiretroviral drug exposure at the time of treatment initiation (ie, women exposed to treatment for the prevention of mother-to-child transmission or defaulters re-initiating first-line ART after a period of treatment interruption) are substantially more likely to have viruses resistant to both NNRTIs and NRTIs compared with people who report being antiretroviral-drug naive, in whom we infer resistance as being due to transmitted drug resistance. Data from several LMICs suggest that this pre-exposed population represents around 10–30% of people initiating or re-initiating first-line NNRTI-containing ART, a proportion that is likely to increase substantially following the rapid expansion in HIV treatment coverage.^1^

The present study also provides the first estimates of the risk of virological failure in individuals with previous exposure to antiretroviral drugs who initiate or re-initiate first-line NNRTI-based regimens in LMICs. The risk of virological failure was almost three times higher in the previously exposed group than in the treatment-naive group, which is reminiscent of data showing that presence of NNRTI resistance mutations before first-line treatment is associated with two to three times higher prevalence of virological failure after 12 months of treatment.^10^, ^12^ These results suggest that self-reported previous exposure might be usable in clinic to identify people at increased risk of treatment failure and resistance. These findings underpin WHO guidelines on pretreatment drug resistance, which recommend the identification of individuals starting ART who are at increased risk of pretreatment drug resistance due to previous antiretroviral exposure (or other risk), with prioritisation of treatment with non-NNRTI based ART in this subpopulation. An alternative approach would be to identify patients with previous exposure and improve monitoring for early virological failure, although the problems of feedback of viral load results to patients and clinicians are likely to be significant in sub-Saharan Africa.

Notably, NRTI resistance also increased significantly over time in eastern and southern Africa. The prevalence of NRTI resistance was substantially lower than that of NNRTI resistance. This finding is not surprising for several reasons. First, in patients with virological failure, NNRTI mutations are among the earliest to emerge^20^ and therefore greater transmission of these resistant variants retaining almost wild-type replication fitness could be expected.^21^ The NRTI mutation Met184Val/Ile also emerges early, but is not commonly transmitted because of its significant fitness cost to the virus. In our analysis, about 10% of patients with pretreatment drug resistance had Met184Val/Ile mutations. Although a sub-analysis showed a significant doubling of the prevalence of Met184Val/Ile in individuals with previous exposure to antiretroviral drugs, this could nevertheless suggest previous exposure even in patients who did not report it.

Thymidine analogue mutations were common, consistent with rapid accumulation of such mutations following the failure of first-line thymidine analogue-containing ART.^22^, ^23^ By contrast, despite the scale-up of tenofovir-based ART and reports of the emergence of resistance to tenofovir in patients with treatment failure on tenofovir across sub-Saharan Africa,^8^ resistance to tenofovir was infrequent in our analysis. This finding is somewhat reassuring given concerns about the use of tenofovir for both the treatment and prevention of HIV infection.^24^ Nonetheless, continued surveillance for Lys65Arg/Asn and associated mutations such as Ala62Val remains important as tenofovir use is relatively new in many LMICs.^25^

Our study reports the prevalence of pretreatment drug resistance among 56 000 people initiating ART over two decades, which we identified via a systematic review that included information from nine nationally representative unpublished pretreatment drug-resistance surveys (n=7138) and five additional unpublished studies (n=2757), making ours the largest such study to date. In addition to adding contemporary data to our previous report,^9^ this analysis includes 2·4 times more data from Latin America and the Caribbean and 2·9 times more data from Asia than in our previous work, allowing us to do statistically robust analyses in these regions, where it was previously unclear whether NNRTI resistance was increasing.

Our study has important limitations including the large amount of between-study heterogeneity and the inclusion of studies that are not nationally representative (appendix). The large degree of unexplained heterogeneity reduces the statistical power to detect region-specific trends over time. The inclusion of non-nationally representative studies has the potential to yield estimates of resistance that are too high or low. However, we were reassured by noting that the results from nationally representative surveys included in our analysis showed estimates of the prevalence of pretreatment drug resistance that were broadly similar to those from studies using a convenience sample in the same countries. In addition to these limitations, there were few studies from rural settings, so the analysis reflects the prevalence of pretreatment drug resistance primarily in urban and peri-urban areas. However, as access to ART has substantially increased in sub-Saharan Africa, similar trends might well be expected in rural areas with similar ART programmes. The inclusion of results using different genotyping methods and interpretation systems is a potential drawback of this analysis. Finally, we did not account for mutations in the connection domain of reverse transcriptase, which is known to confer NRTI and NNRTI resistance with mutations such as Asn348Ile;^26^, ^27^ therefore, the prevalence of pretreatment drug resistance might have been underestimated.

With increasing global use of ART for both the treatment and prevention of HIV infection and increasing global trends in the prevalence of HIV drug resistance, efforts to improve HIV programme quality and prevent the emergence and transmission of drug-resistant HIV should be strengthened. Although, HIV drug-resistance testing is not routinely available for individual patient management in many LMICs, the monitoring of patient and clinical factors associated with the emergence of preventable HIV drug resistance and successful population-level viral load suppression are relatively inexpensive, and can be used to identify gaps in service delivery and programme quality that can be corrected. These factors include retention on ART 12 months after treatment initiation, on-time pill pick-up, drug stock outs, viral load suppression, viral load completion, and timely switching to second-line ART. Monitoring of these factors, or early warning indictors of HIV drug resistance, is recommended by WHO on an annual basis at all ART clinics through the implementation of the consolidated strategic information guidelines for HIV in the health sector.^28^, ^29^, ^30^

Our findings reinforce the need for routine, robust nationally representative surveillance of pretreatment drug resistance and the need for each country to assess the prevalence of pretreatment drug resistance in people starting ART, irrespective of reported previous exposure to antiretroviral drugs. WHO guidelines provide recommendations on the appropriate public health response in the face of rising prevalence of pretreatment NNRTI resistance. The data presented in this report will help to inform policy makers and donors on the urgent need to respond to pretreatment drug resistance in settings where a high prevalence of resistance has already been reached.

At present, point-of-care drug resistance testing is not available, although substantial efforts and resources have been directed to this field and candidate signature mutations for surveillance have been identified.^31^ Earlier treatment initiation combined with routine viral load monitoring in people on treatment should lead to more viral load suppression and less pretreatment drug resistance, providing that the people who are identified as having confirmed virological failure are promptly switched to active second-line and or third-line regimens. One important caveat, however, is the fact that individuals on first line treatment sometimes experience virological rebound with subsequent re-suppression even in the absence of viral load monitoring,^32^ reinforcing the continual need for adherence support. Particular attention should be paid to people initiating ART who have had previous exposure to antiretroviral drugs, given that our data show increased prevalence of not only NNRTI but also NRTI mutations in this group compared with antiretroviral drug-naive individuals as well as an independently higher risk of virological failure. Finally, routine surveillance of population-level HIV drug resistance coupled with strengthened approaches to prevent the development of resistance and sound evidence-based responses will be essential to the attainment of the global goal to eliminate AIDS as a public health threat by 2030.

For the **Stanford HIVdb algorithm** see https://hivdb.stanford.edu/

For the **International Antiviral Society-USA mutations list** see https://www.iasusa.org/content/drug-resistance-mutations-in-HIV

For the **Agence autonome de l'Inserm algorithms** see http://www.hivfrenchresistance.org/

**This online publication has been corrected. The corrected version first appeared at thelancet.com/infection on December 15, 2017**
