# Corrections

**DOI:** 10.1016/S1473-3099(17)30749-1

**Published:** 2018-02

*HIV-1 drug resistance before initiation or re-initiation of first-line antiretroviral therapy in low-income and middle-income countries: a systematic review and meta-regression analysis.* Lancet Infect Dis *2017; published online Nov 31. http://dx.doi.org/10.1016/S1473-3099(17)30702-8*—In this Article, the text in parentheses in the first sentence of the third paragraph of the Data analysis section has been amended to read “calendar year at the midpoint of the study year”; the y-axis of figure 2 has been corrected to read “Prevalence of NRTI resistance (%)”; and the legend of figure 3 has been updated to include a definition of NNRTI. These corrections have been made to the online version as of Dec 15, 2017.

