# Supplementary material for: HIV-1 drug resistance before initiation or re-initiation of first-line antiretroviral therapy in low-income and middle-income countries: a systematic review and meta-regression analysis
Source: Lancet Infect Dis. 2018 Mar;18(3):346–55. doi: 10.1016/S1473-3099(17)30702-8 (PMC5835664; doi:10.1016/S1473-3099(17)30702-8)
Supplement: Supplementary appendix [file mmc1.pdf]

THE LANCET
Infectious Diseases

Supplementary appendix

This appendix formed part of the original submission and has been peer reviewed. We post it as supplied by the authors.

Supplement to: Gupta R K, Gregson J, Parkin N, et al. HIV-1 drug resistance before initiation or re-initiation of first-line antiretroviral therapy in low-income and middle-income countries: a systematic review and meta-regression analysis. *Lancet Infect Dis* 2017; published online Nov 30. http://dx.doi.org/10.1016/S1473-3099(17)30702-8.

Supplementary Figure 1: Flow chart of included datasets

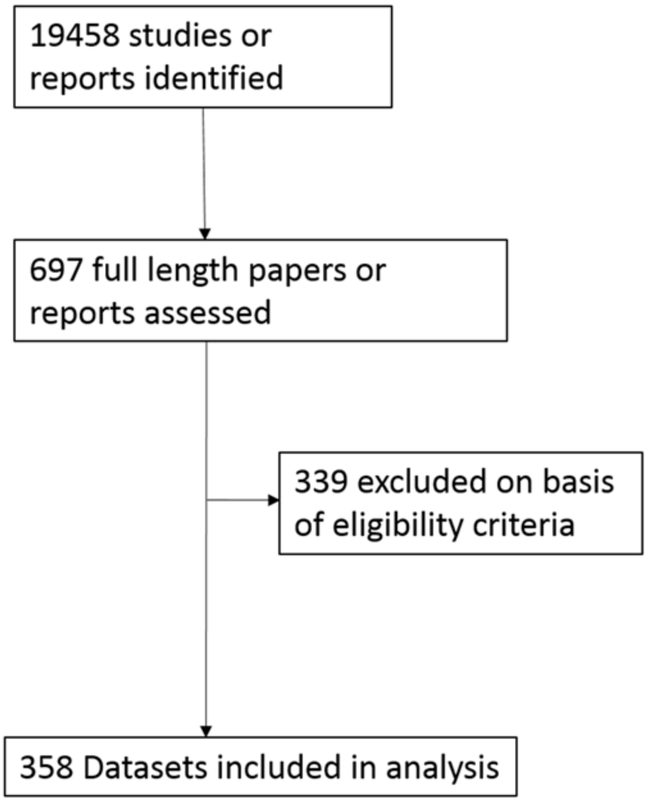

Supplementary Table 1: List of included studies in Asia

Study	Country	Mid-point year	Number of patients genotyped	Urban or rural?
Ly	Cambodia	2003.5	136	Urban
Nouhin	Cambodia	2006.5	67	Urban
WHO survey	Cambodia	2008	43	Urban
Han Zhang	China	2001.5	91	Urban
Zhang	China	2004	40	Urban
Liao	China	2004.5	676	Urban
Liu	China	2004.5	52	Urban
Zhong	China	2005	95	Urban
Tu	China	2005.5	49	Urban
Han Wang	China	2006	80	Urban
Liu Lu	China	2006	21	Urban
Zhang Kang	China	2006	53	Urban
Chin	China	2006.5	32	Urban
Chunfu Yang	China	2006.5	47	Urban
WHO survey	China	2007	44	Urban
WHO survey	China	2008	234	Urban
Zeng 2013	China	2008	159	Urban
Taser-S Jiamsakul	China	2008.5	88	Urban
Li Yijia	China	2009	379	Urban
WHO survey	China	2009	348	Urban
Li Lu	China	2009.5	21	Urban
Chen	China	2010	299	Urban
Guo 2015	China	2010	150	Urban
Li 2014	China	2010	98	Urban
Yang 2012	China	2010	119	Urban
Yang 2013	China	2010	489	Urban
Zou 2013	China	2010	90	Urban
Chen 2014	China	2011	131	Urban
Li 2013	China	2011	94	Urban
Wang X 2012	China	2011	627	Urban
Jiao 2014	China	2012	223	Urban
Lu 2016	China	2013	152	Urban
Zhang 2015	China	2013	211	.
Eshleman	India	1999.5	12	Urban
Balakrishnan	India	2002.5	50	Urban
Deshpande	India	2003	128	Urban
Iqbal 2011	India	2006	18	Urban
Kandathil	India	2006	93	Urban
Chaturburj	India	2007	34	Urban
Hingankar Ind-Pms-2007-Mum	India	2007	139	.
Lall	India	2007	40	Urban
Sinha	India	2007	68	Urban
Rajesh	India	2007.5	104	Urban
Hingankar Ind-Pms-2008-Che	India	2008	147	.
Neogi	India	2009.5	21	Urban

Study	Country	Mid-point year	Number of patients genotyped	Urban or rural?
Neogi	India	2010	270	.
Thorat	India	2010	47	Urban
Kannangai	India	2010.5	127	Urban
Azam	India	2011	44	.
Neogi	India	2011	170	Urban
Shet	India	2011	599	Urban
WHO survey	Indonesia	2006	47	Urban
Jahanbakhsh	Iran	2010.5	47	Urban
Baesi	Iran	2011	30	Urban
Memarnejadian	Iran	2011	40	Urban
Mokhbat	Lebanon	2007	37	Urban
Ong	Malaysia	2009	100	Urban
WHO survey	Myanmar	2016	327	.
Bhusal	Nepal	2011	15	Urban
Shah	Pakistan	2008	31	Urban
Gare	Papua New Guinea	2010	96	Urban
Lavu	Papua New Guinea	2013.5	123	Urban
Chen	Taiwan	2010	301	.
Auswinporn	Thailand	2000.5	21	Urban
Tovanabutra	Thailand	2000.5	34	Urban
Chalermchockcharoenkit	Thailand	2002	34	Urban
Chonwattana	Thailand	2002.5	165	Urban
Apisarnthanarak	Thailand	2004.5	305	Urban
Lallemant	Thailand	2005	444	Urban
WHO survey	Thailand	2005	145	Urban
Sirivichayakul	Thailand	2005.5	92	.
Apisarnthanarak	Thailand	2006	151	Urban
Auwanit	Thailand	2006	50	Urban
Taser-S Jiamsa	Thailand	2008	17	Urban
Sungkanuparph	Thailand	2008.5	466	Urban
Ayouba	Thailand	2009	56	Urban
Colbn	Thailand	2009	184	Urban
Sirivichayakul	Thailand	2009	284	Urban
Ananworanich	Thailand	2011	120	Urban
Manosuthi	Thailand	2011	330	Urban
Sungkanuparph	Thailand	2011	50	Urban
Sayan	Turkey	2012.5	1306	Urban
Nguyen	Vietnam	2006	49	Urban
Thao Vu	Vietnam	2006	219	Urban
Ishizaki	Vietnam	2007	272	Urban
Phan	Vietnam	2008	173	Urban
Tanuma	Vietnam	2008	292	Urban
WHO survey	Vietnam	2008	73	Urban
Bontell	Vietnam	2008.5	63	Urban
Dean	Vietnam	2008.5	92	Urban
Ayouba	Vietnam	2009	63	Urban

Study	Country	Mid-point year	Number of patients genotyped	Urban or rural?
Ishizaki	Vietnam	2009	139	Urban
Tanuma	Vietnam	2009	250	Urban
Pham	Vietnam	2009.5	490	.
Tanuma	Vietnam	2010	292	Urban
Tanuma	Vietnam	2011	294	Urban
Tanuma	Vietnam	2012	261	Urban

Supplementary Table 2: List of included studies in Eastern Africa. Pms studies are PASER[1]

Study	Country	Mid-point year	Number of patients genotyped	Urban or rural?
Vidal	Burundi	2002	119	Urban
Nzeyimana	Burundi	2007.5	116	Urban
Kassau	Ethiopia	2003	92	Urban
Abegaz	Ethiopia	2005	39	Urban
Huruy	Ethiopia	2010	83	Urban
Nafisa	Kenya	2004	398	Rural
Lihana	Kenya	2005	475	Urban
WHO survey	Kenya	2005	38	Urban
Kantor 2014	Kenya	2006	55	Rural
Kiptoo 2013	Kenya	2006	188	Rural
Hamers Ken-Pms-	Kenya	2007	210	.
Price	Kenya	2007.5	64	Urban
Hamers Ken-Pms-	Kenya	2008	206	.
Hassan 2013	Kenya	2009	182	Rural
Lihana	Kenya	2009	100	Urban
Sigaloff	Kenya	2010	68	Urban
Uwash	Kenya	2010	169	.
Hassan 2016	Kenya	2011	50	Rural
Frenkel 2017	Kenya	2014	517	.
Servais	Rwanda	2000	43	Urban
Price	Rwanda	2007.5	78	Urban
Rusine 2013	Rwanda	2008	109	Urban
Mutagoma 2016	Rwanda	2011	57	Urban
Mosha	Tanzania	2004.5	75	Urban
Nyombi	Tanzania	2005	100	Rural
Masimba 2013	Tanzania	2006	120	Rural
WHO survey	Tanzania	2006	50	Urban
Chunfu Yang	Tanzania	2006.5	49	Urban
Kasang 2011	Tanzania	2009	88	.
Masimba 2013	Tanzania	2009	119	Rural
Vairo 2013	Tanzania	2010	67	Urban
Becker-Pergola	Uganda	1993.5	27	Urban
Weidle	Uganda	1997	11	Urban
Eshleman	Uganda	1998	12	Urban
Gale	Uganda	1999.5	187	Rural
Eshleman Aids	Uganda	2000.5	104	Urban
Church	Uganda	2001.5	203	Urban
Galluzzo	Uganda	2003	88	Urban
Lyagoba	Uganda	2003.5	93	Urban
Lee Croi	Uganda	2006.5	401	Rural
Ndembi	Uganda	2006.5	37	Urban
Ssemwanga 2012	Uganda	2007	72	Rural
Price	Uganda	2007.5	92	Urban
Hamers Uga-Pms-2008-Jcr 2011	Uganda	2008	194	.
Hamers Uga-Pms-2008-Jfp 2011	Uganda	2008	202	.

Study	Country	Mid-point year	Number of patients genotyped	Urban or rural?
Hamers Uga-Pms-2008-Mba 2011	Uganda	2008	210	.
Kityo	Uganda	2008	111	Urban
Ssemwanga D	Uganda	2009	42	Urban
Ndembi	Uganda	2009.5	70	Urban
Nazziwa J; Ndembi N	Uganda	2010	47	Urban
Reynolds 2016	Uganda	2012.5	75	Rural
Kaleebu 2015	Uganda	2013	425	Urban
WHO survey	Uganda	2016	342	.

Supplementary Table 3: List of included studies in Latin America

Study	Country	Mid-point year	Number of patients genotyped	Urban or rural?
Kijak	Argentina	1997	52	.
Kijak	Argentina	1998.5	86	Urban
Pando	Argentina	2001	16	Urban
Dilernia	Argentina	2004	284	.
Petroni	Argentina	2004.5	52	Urban
Pando	Argentina	2007	214	Urban
Rodriguez-Rodriguez	Argentina	2011	197	Urban
Cecchini	Argentina	2012	91	Urban
Bissio Arg-Pdr-2017	Argentina	2014	294	.
Teixeira	Brazil	1995.5	27	Urban
Dumans	Brazil	1998	47	Urban
Barreto	Brazil	2000	335	.
Sucupira	Brazil	2000	90	.
Teixeira	Brazil	2000	38	Urban
Varella	Brazil	2000	71	.
Brindeiro	Brazil	2001	409	Urban
Pires	Brazil	2001	50	Urban
Sa-Ferreira	Brazil	2002	74	Urban
De Madeiros	Brazil	2002.5	84	Urban
Eyer-Silva	Brazil	2003	27	Urban
Tupinambas	Brazil	2003	64	Urban
Gonsalez	Brazil	2004	123	Urban
Rodrigues	Brazil	2004	108	Urban
Sanabani	Brazil	2004	50	Urban
Eyer-Silva	Brazil	2005	50	Urban
Ferreira	Brazil	2005.5	57	Urban
Brigido	Brazil	2006	193	Urban
De Medeiros	Brazil	2006	99	Urban
Ferreira	Brazil	2006	49	Urban
Cardoso	Brazil	2007	95	Urban
De Sa Filho	Brazil	2007	33	Urban
Pilotto	Brazil	2007	197	Urban
Santos	Brazil	2007	85	.
Sprinz	Brazil	2007	375	Urban
Cavalcanti	Brazil	2008	130	Urban
Couto-Fernandez	Brazil	2008	299	Rural
Da Silveira	Brazil	2008	48	Urban
Inocencio	Brazil	2008	210	Urban
Arruda	Brazil	2008.5	63	Urban
Bacelar Acioli Lins	Brazil	2008.5	53	Urban
Carvalho	Brazil	2008.5	52	Urban
Ferreira	Brazil	2008.5	92	Urban
Graf	Brazil	2008.5	82	Urban
Soares	Brazil	2008.5	251	Urban
Alencar	Brazil	2009	287	.

Study	Country	Mid-point year	Number of patients genotyped	Urban or rural?
Bermudez-Aza	Brazil	2009	99	Urban
Ferreira	Brazil	2009	221	.
Gaspareto	Brazil	2009	48	Urban
Prellwitz	Brazil	2009	31	Urban
De Moraes	Brazil	2010	329	Urban
Da Costa	Brazil	2011	18	Urban
De Lourdes Teixeira 2015	Brazil	2011	229	.
Teixeira	Brazil	2011	173	Urban
Velasco De Castro 2014	Brazil	2011	246	.
Soares Moura 2015	Brazil	2012	106	Rural
De Souza-Guimaraes	Brazil	2013	186	Urban
WHO survey	Brazil	2013	1391	.
Diazgranados	Colombia	2007.5	103	Urban
WHO survey	Colombia	2016	192	.
Ruibal-Brunet	Cuba	1999	27	Urban
Perez	Cuba	2003	250	Urban
Perez	Cuba	2009	152	Urban
Machado	Cuba	2010	183	.
Perez 2014	Cuba	2010	323	Urban
Myers	Dominican Republic	2009	103	Urban
Murillo	El Salvador	2008	119	.
Holguin	El Salvador	2010	61	.
Avila Rios 2011	Guatemala	2010.5	145	Urban
Avila-Rios	Guatemala	2011.5	1084	Urban
WHO survey	Guatemala	2015	241	.
Lloyd	Honduras	2002.5	336	Urban
Murillo	Honduras	2005.5	200	Urban
Avila-Rios 2015	Honduras	2014	365	Urban
Hamilton	Jamaica	2009	72	Urban
Barrow	Jamaica	2011	79	Urban
Valle-Bahena	Mexico	2002	36	Urban
Escoto-Delgadillo	Mexico	2002.5	96	Urban
Viani	Mexico	2004	41	Urban
WHO survey	Mexico	2004	47	Urban
Avila-Rios	Mexico	2007.5	1655	Urban
Avila-Rios Mex-Pdr-2015 2015	Mexico	2015	260	.
Avila-Rios 2016	Nicaragua	2013	283	Urban
WHO survey	Nicaragua	2016	171	.
Ahumada-Ruiz	Panama	2004.5	53	Urban
Lama	Peru	2002.5	359	.
Soria	Peru	2008	36	.
Delgado	Venezuela	1998.5	31	Urban

Study	Country	Mid-point year	Number of patients genotyped	Urban or rural?
Bouchard	Venezuela	2003	20	Urban
Rangel	Venezuela	2005.5	63	Urban
Castillo	Venezuela	2008	62	Urban

Supplementary Table 4: List of included studies in Southern Africa. Pms studies are PASER[1]

Study	Country	Mid-point year	Number of patients genotyped	Urban or rural?
WHO survey	Botswana	2005	127	Urban
Chunfu Yang	Botswana	2006.5	70	Urban
Rowley 2016	Botswana	2012	220	.
Rowley 2016	Botswana	2013.5	140	.
Rowley 2016	Botswana	2014.5	62	.
WHO survey	Lesotho	2009	38	Urban
Petch	Malawi	1998.5	21	Urban
Church	Malawi	2001	40	Urban
Kamoto	Malawi	2006	34	Urban
Chunfu Yang	Malawi	2006.5	53	Urban
Wadonda-Kabondo N 2012	Malawi	2008	579	Urban
WHO survey	Malawi	2009	102	Urban
Lahuerta	Mozambique	1999	51	Urban
Abreu	Mozambique	2002	75	Urban
Ferreira	Mozambique	2002	47	Urban
Bartolo	Mozambique	2003	104	Urban
Bellocchi	Mozambique	2003	58	Urban
Perreira	Mozambique	2003	43	Urban
Lahuerta	Mozambique	2004	51	Urban
WHO survey	Mozambique	2007	76	Urban
WHO survey	Mozambique	2009	112	Urban
Bila 2015	Mozambique	2009.5	68	Urban
Ruperez 2016	Mozambique	2011	150	Rural
WHO survey	Namibia	2015	383	.
Pillay	South Africa	2000	37	Urban
Gordon Jv	South Africa	2000.5	56	Urban
Pillay	South Africa	2002	65	Urban
Bessong	South Africa	2002.5	40	Rural
Jacobs	South Africa	2003	140	Urban
Pillay	South Africa	2004	48	Urban
Orrell	South Africa	2004.5	120	Urban
Barth	South Africa	2005	313	Urban
Mcintyre	South Africa	2005	393	Urban
Huang	South Africa	2006	397	Urban
Van Zyl	South Africa	2006	97	Urban
Hamers Zaf-Pms-2007-Acc 2011	South Africa	2007	211	.
Hamers Zaf-Pms-2007-Mmh 2011	South Africa	2007	196	.
Hamers Zaf-Pms-2007-Tlc 2011	South Africa	2007	183	.
Bessong	South Africa	2008	80	Rural
Nwobegahay 2012	South Africa	2008	93	Urban
Mavhandu	South Africa	2008.5	58	Rural
Jacobs 2014	South Africa	2009	73	Urban

Study	Country	Mid-point year	Number of patients genotyped	Urban or rural?
Parboosing	South Africa	2009	47	Urban
Wela Msimanga 2015	South Africa	2009	41	Rural
Manasa 2016	South Africa	2011	701	Rural
Derache 2016	South Africa	2013	185	.
Chimukangara	South Africa	2014	1719	.
Steegen 2016	South Africa	2014	277	Urban
Gillian Hunt	South Africa	2016	303	Urban
Maphalala	Swaziland	2006	61	Urban
WHO survey	Swaziland	2009	38	Urban
Handema	Zambia	2000	28	Urban
Hamers Zmb-Pms-2007-Chc 2011	Zambia	2007	219	.
Hamers Zmb-Pms-2007-Kar 2011	Zambia	2007	208	.
Hamers Zmb-Pms-2007-Lth 2011	Zambia	2007	104	.
Price	Zambia	2007.5	169	Urban
Lyagoba Dunn	Zimbabwe	2003.5	173	Urban
Tshabalala	Zimbabwe	2006.5	236	Urban
Hamers Zwe-Pms-2008-Con 2011	Zimbabwe	2008	206	.
Mungati 2016	Zimbabwe	2009	1483	Urban
WHO survey	Zimbabwe	2015	353	.

Supplementary Table 5: List of included studies in Western/Central Africa. Pms studies are PASER[1]

Study	Country	Mid-point year	Number of patients genotyped	Urban or rural?
Bartolo	Angola	2001	122	Urban
Ferreira	Angola	2004	44	Urban
Castelbranco	Angola	2008.5	35	Urban
Alonso 2012	Angola	2009	101	Urban
Bartolo 2014	Angola	2009	139	Urban
WHO survey	Angola	2009	39	Urban
Chamberland 2012	Benin	2009	129	Urban
Vergne	Burkina Faso	2003	97	Urban
Tebit	Burkina Faso	2005	104	Urban
Ayouba	Burkina Faso	2009	51	Urban
WHO survey	Burkina Faso	2009	48	Urban
Aghokeng	Cameroon	1998	49	Urban
Vergne	Cameroon	2001.5	53	Urban
Aghokeng	Cameroon	2002	50	Urban
Vessiere	Cameroon	2002.5	96	Urban
Soares	Cameroon	2003.5	59	Urban
Koizumi	Cameroon	2004	51	Rural
Ndembi	Cameroon	2004	76	Urban
Aghokeng	Cameroon	2006.5	217	.
Burda Jmv	Cameroon	2006.5	21	Urban
Aghokeng	Cameroon	2007	81	Urban
Billong Cmr-Pms-2009-Yao 2013	Cameroon	2009	137	.
Nanfack 2016	Cameroon	2013	66	.
Fokam 2016	Cameroon	2014	49	Urban
Cameroon Who Survey	Cameroon	2015	252	.
Oliveira	Cape Verde	2006	18	Urban
Marechal	Central African Republic	2005	140	Urban
Aghokeng	Chad	2006.5	34	Urban
Bruzzone	Congo	2007	74	Rural
Toni	Cote d'Ivoire	1998.5	99	Urban
Toni	Cote d'Ivoire	2001.5	107	Urban
Adje-Toure	Cote d'Ivoire	2002	20	Urban
Toni	Cote d'Ivoire	2004	100	Urban
Ayouba	Cote d'Ivoire	2007	48	Urban
Vidal	Democratic Republic of the Congo	2002	70	Urban
Djoko	Democratic Republic of the Congo	2007	94	Urban
Muwonga 2011	Democratic Republic of the Congo	2008	253	Urban

Study	Country	Mid-point year	Number of patients genotyped	Urban or rural?
Kamangu 2015	Democratic Republic of the Congo	2013.5	130	Urban
Caron 2012	Gabon	2007	107	Urban
Bonney 2013	Ghana	2008	53	Urban
Nii-Trebi 2013	Ghana	2009	59	Urban
Charpentier 2011	Guinea-Conakry	2009	94	Urban
Derache	Mali	2005	98	Urban
Haidara	Mali	2007.5	100	Urban
Issiaka Maiga 2013	Mali	2010	51	Urban
Mamadou 2009	Niger	2011	96	Urban
Agwale	Nigeria	2006.5	18	Urban
Ajoge 2012	Nigeria	2007	28	Urban
Hamers Nga-Pms-2008-Lut 2011	Nigeria	2008	195	.
WHO survey	Nigeria	2008	271	Urban
Imade 2014	Nigeria	2011	29	.
Diop-Ndiaye	Senegal	1999.5	96	Urban
Vergne	Senegal	1999.5	41	Urban
Diop-Ndiaye	Senegal	2005	104	Urban
Ayouba	Senegal	2009	48	Urban
Yaotse	Togo	2006.5	83	Urban

Table S6: **Estimates of heterogeneity,** as assessed by I^2 statistic, within regions. P-values are for heterogeneity*

Class	Region	Overall (all calendar years)
Any	Asia	I^2=72.5%; P<0.0001
	Eastern Africa	I^2=84.7%; P<0.0001
	Latin America	I^2=72.5%; P<0.0001
	Southern Africa	I^2=86.1%; P<0.0001
	Western/Central Africa	I^2=66.0%; P<0.0001
NNRTI	Asia	I^2=55.2%; P<0.0001
	Eastern Africa	I^2=81.6%; P<0.0001
	Latin America	I^2=78.2%; P<0.0001
	Southern Africa	I^2=86.2%; P<0.0001
	Western/Central Africa	I^2=54.9%; P<0.0001
NRTI	Asia	I^2=50.2%; P<0.0001
	Eastern Africa	I^2=63.5%; P<0.0001
	Latin America	I^2=66.7%; P<0.0001
	Southern Africa	I^2=66.6%; P<0.0001
	Western/Central Africa	I^2=57.4%; P<0.0001

*P-value is a test of whether there is at least some between study heterogeneity. Estimates of I^2 are derived from meta-analyses using the Freeman Tukey arcsin transformation as described in the Supplementary Methods

Table S7: Proportion of patients with pre-treatment drug resistance by study-level proportion of female patients

	Region	P-value	<50% female		At least 50% female		Unknown	
			Studies	Proportion (95% CI)	Studies	Proportion (95% CI)	Studies	Proportion (95% CI)
Any	Asia	0.0093	38	4.7 (3.7 to 5.9)	13	2.2 (1.3 to 3.9)	42	3.5 (2.6 to 4.5)
	Eastern Africa	0.5121	6	3.6 (1.9 to 7.0)	24	2.7 (1.7 to 4.3)	22	4.4 (2.7 to 7.1)
	Latin America	0.5464	10	7.9 (5.5 to 11.2)	3	10.0 (5.5 to 17.7)	77	8.2 (7.2 to 9.3)
	Southern Africa	0.7319	4	2.8 (1.8 to 4.5)	34	3.0 (2.0 to 4.5)	22	3.4 (2.2 to 5.2)
	Western/Central Africa				17	3.6 (2.2 to 5.8)	36	3.9 (2.8 to 5.4)
NNRTI	Asia	0.1268	40	1.8 (1.4 to 2.5)	13	0.9 (0.3 to 2.4)	39	1.8 (1.2 to 2.6)
	Eastern Africa	0.2692	6	2.4 (1.2 to 4.6)	25	1.0 (0.5 to 2.3)	22	3.1 (1.9 to 5.0)
	Latin America	0.7542	10	3.8 (2.0 to 7.0)	3	4.8 (1.8 to 12.1)	77	3.3 (2.6 to 4.1)
	Southern Africa	0.4314	4	1.4 (0.7 to 2.7)	34	1.9 (1.1 to 3.1)	22	2.1 (1.2 to 3.8)
	Western/Central Africa				17	2.0 (1.0 to 3.9)	37	1.6 (1.0 to 2.5)
NRTI	Asia	0.7697	40	1.4 (1.0 to 1.9)	13	1.5 (0.8 to 2.9)	41	1.0 (0.6 to 1.8)
	Eastern Africa	0.2803	6	0.7 (0.3 to 1.5)	25	1.1 (0.6 to 1.9)	21	1.9 (1.1 to 3.3)
	Latin America	0.4861	10	3.1 (2.0 to 4.9)	3	4.9 (3.5 to 6.9)	77	3.9 (3.2 to 4.7)
	Southern Africa	0.2713	4	1.6 (0.8 to 3.1)	35	0.6 (0.3 to 1.3)	22	0.7 (0.4 to 1.4)
	Western/Central Africa				18	1.7 (0.9 to 3.4)	38	1.6 (1.0 to 2.5)

P-value is a test for heterogeneity across categories, excluding data where the exposure is unknown

Table S8: Proportion of patients with pre-treatment drug resistance by study-level mean/median CD4 count

	Region	P-value	CD4 <=300 cells/mm3		CD4 >300 cells/mm3		Unknown CD4 count	
			Studies	Proportion (95% CI)	Studies	Proportion (95% CI)	Studies	Proportion (95% CI)
Any	Asia	0.9820	20	4.3 (3.1 to 5.9)	16	4.4 (3.0 to 6.4)	57	3.4 (2.6 to 4.3)
	Eastern Africa	0.8896	7	5.0 (3.3 to 7.4)	10	5.0 (3.0 to 8.3)	35	2.6 (1.6 to 4.2)
	Latin America	0.4317	6	9.9 (7.6 to 12.9)	15	8.0 (6.1 to 10.3)	69	8.1 (7.0 to 9.3)
	Southern Africa	0.7578	9	4.0 (2.7 to 5.8)	9	3.9 (1.9 to 7.9)	42	2.7 (1.8 to 4.0)
	Western/Central Africa	0.0356	13	4.3 (2.7 to 6.7)	14	1.8 (0.9 to 3.7)	26	5.2 (3.7 to 7.1)
NNRTI	Asia	0.9763	21	1.9 (1.3 to 2.7)	16	2.2 (1.5 to 3.2)	55	1.6 (1.1 to 2.2)
	Eastern Africa	0.7215	7	3.1 (1.8 to 5.3)	10	2.5 (1.2 to 4.9)	36	1.6 (0.9 to 2.8)
	Latin America	0.2737	6	6.6 (5.3 to 8.1)	15	3.9 (2.6 to 5.8)	69	3.1 (2.4 to 4.0)
	Southern Africa	0.4390	9	1.9 (0.8 to 4.3)	9	2.9 (1.3 to 6.4)	42	1.8 (1.1 to 2.8)
	Western/Central Africa	0.0716	14	2.5 (1.5 to 4.4)	14	0.8 (0.2 to 2.6)	26	1.9 (1.1 to 3.1)
NRTI	Asia	0.2418	21	1.6 (0.9 to 2.7)	16	1.1 (0.5 to 2.0)	57	1.3 (1.0 to 1.8)
	Eastern Africa	0.7239	7	2.1 (1.3 to 3.4)	10	1.6 (0.7 to 3.5)	35	0.9 (0.5 to 1.7)
	Latin America	0.0841	6	5.1 (3.2 to 8.0)	15	3.0 (2.0 to 4.3)	69	3.9 (3.2 to 4.7)
	Southern Africa	0.2072	9	1.2 (0.6 to 2.4)	9	0.5 (0.1 to 1.8)	43	0.7 (0.4 to 1.2)
	Western/Central Africa	0.1054	15	1.5 (0.7 to 3.3)	14	0.7 (0.2 to 1.9)	27	2.5 (1.7 to 3.8)

P-value is a test for heterogeneity across categories, excluding data where the exposure is unknown

Table S9: Proportion of patients with pre-treatment drug resistance by study-level proportion of men who have sex with men

	Region	P-value	<50% MSM		At least 50% MSM		Unknown	
			Studies	Proportion (95% CI)	Studies	Proportion (95% CI)	Studies	Proportion (95% CI)
Any	Asia	0.0472	62	3.3 (2.6 to 4.1)	14	5.3 (3.7 to 7.6)	17	4.5 (3.1 to 6.5)
	Eastern Africa	0.4749	34	2.2 (1.4 to 3.4)	1	5.0 (1.6 to 11.3)	17	7.0 (5.1 to 9.6)
	Latin America	0.0672	42	8.4 (7.3 to 9.7)	5	5.6 (4.1 to 7.5)	43	8.2 (6.8 to 9.9)
	Southern Africa	.	40	2.0 (1.3 to 3.0)	.		20	5.5 (4.3 to 7.1)
	Western/Central Africa	.	45	3.6 (2.7 to 5.0)	.		8	4.5 (2.6 to 7.7)
NNRTI	Asia	0.7164	64	1.6 (1.2 to 2.0)	13	1.8 (1.1 to 2.9)	15	2.3 (1.2 to 4.6)
	Eastern Africa	0.2836	34	1.0 (0.6 to 1.8)	1	4.0 (1.1 to 9.9)	18	4.9 (3.5 to 6.9)
	Latin America	0.3832	42	3.0 (2.2 to 4.1)	5	2.0 (0.9 to 4.8)	43	4.1 (3.1 to 5.4)
	Southern Africa	.	41	1.0 (0.6 to 1.8)	.		19	4.2 (3.1 to 5.8)
	Western/Central Africa	.	44	1.3 (0.8 to 2.1)	.		10	3.8 (2.4 to 5.9)
NRTI	Asia	0.9647	65	1.2 (0.8 to 1.6)	13	1.2 (0.6 to 2.4)	16	2.2 (1.4 to 3.4)
	Eastern Africa	0.9644	34	0.9 (0.6 to 1.5)	1	1.0 (0.0 to 5.4)	17	2.5 (1.5 to 4.1)
	Latin America	0.2158	42	4.1 (3.2 to 5.2)	5	2.9 (1.9 to 4.3)	43	3.7 (2.9 to 4.7)
	Southern Africa	.	41	0.3 (0.1 to 0.9)	.		20	1.2 (0.8 to 1.7)
	Western/Central Africa	.	45	1.4 (0.9 to 2.3)	.		11	2.2 (1.1 to 4.2)

P-value is a test for heterogeneity across categories, excluding data where the exposure is unknown

Table S10: Prevalence of HIV drug resistance by region and by grouped calendar years. P-value is a test for trend across categories of calendar year.

	Region	P-value	Before 2005		2005-9		2010-13		2014-6	
			Studies	Proportion (95% CI)	Studies	Proportion (95% CI)	Studies	Proportion (95% CI)	Studies	Proportion (95% CI)
Any	Asia	0.0138	13	2.6 (1.4 to 4.9)	50	3.4 (2.7 to 4.3)	29	4.8 (3.6 to 6.4)	1	5.5 (3.3 to 8.6)
Any	Eastern Africa	<0.0001	13	0.8 (0.2 to 3.1)	29	3.8 (2.6 to 5.3)	8	7.0 (5.0 to 9.7)	2	11.8 (9.8 to 14.1)
Any	Latin America	<0.0001	31	5.4 (4.0 to 7.3)	36	8.5 (7.5 to 9.7)	17	10.7 (9.2 to 12.5)	6	12.4 (9.2 to 16.6)
Any	Southern Africa	<0.0001	18	1.3 (0.5 to 3.4)	31	2.9 (2.2 to 3.9)	5	5.0 (3.6 to 6.9)	6	12.2 (9.7 to 15.1)
Any	Western/Central Africa	0.7521	17	4.0 (2.6 to 6.0)	32	3.5 (2.3 to 5.2)	3	6.2 (3.5 to 10.9)	1	4.1 (0.5 to 14.0)
NNRTI	Asia	0.1250	12	0.7 (0.2 to 2.8)	50	1.7 (1.3 to 2.3)	29	1.9 (1.3 to 2.7)	1	4.0 (2.1 to 6.7)
NNRTI	Eastern Africa	<0.0001	13	0.1 (0.0 to 2.4)	29	2.2 (1.5 to 3.3)	9	5.3 (3.9 to 7.2)	2	10.1 (8.2 to 12.4)
NNRTI	Latin America	<0.0001	31	0.7 (0.3 to 1.6)	36	3.9 (3.3 to 4.7)	17	6.4 (5.4 to 7.6)	6	8.8 (6.2 to 12.4)
NNRTI	Southern Africa	<0.0001	18	0.9 (0.3 to 2.5)	32	1.9 (1.3 to 2.7)	4	2.9 (1.6 to 5.2)	6	10.7 (8.4 to 13.7)
NNRTI	Western/Central Africa	0.0044	17	1.0 (0.5 to 2.2)	31	1.8 (1.1 to 2.9)	4	5.6 (3.5 to 8.8)	2	5.3 (3.3 to 8.5)
NRTI	Asia	0.9538	13	1.4 (0.6 to 3.1)	51	1.4 (1.0 to 2.0)	29	1.2 (0.7 to 2.0)	1	1.5 (0.5 to 3.5)
NRTI	Eastern Africa	0.1139	13	0.5 (0.1 to 2.2)	29	1.4 (0.8 to 2.3)	9	1.9 (0.9 to 4.1)	1	3.2 (1.6 to 5.7)
NRTI	Latin America	0.9445	31	3.9 (2.7 to 5.4)	36	3.8 (3.0 to 4.8)	17	3.7 (2.5 to 5.5)	6	4.1 (2.5 to 6.5)
NRTI	Southern Africa	0.0351	18	0.4 (0.1 to 2.4)	32	0.6 (0.3 to 1.0)	5	1.4 (0.6 to 3.2)	6	2.2 (1.2 to 3.8)
NRTI	Western/Central Africa	0.3081	17	2.0 (1.3 to 2.9)	32	1.2 (0.6 to 2.3)	5	3.1 (1.3 to 7.5)	2	3.7 (2.0 to 6.5)

P-value is a test for heterogeneity across categories

*Estimates marked with a star use the Freeman-Tukey arcsine transformation, because mixed models did not converge, and so could not provide an estimated prevalence.

Table S11: Studies with HIV drug resistance data in both populations with and without reported prior exposure to ARV drugs prior to first-line ART initiation

				Genotyped		Any WHO SDRM		NNRTI		NRTI	
**Region**	Country	Study	Midpoint Year	Pre-treated	Naive	Pre-treated	Naive	Pre-treated	Naive	Pre-treated	Naive
**Asia**	India	Hingankar 2012	2007	10	129	4	7	4	7	3	2
**Asia**	India	Hingankar 2012	2008	4	143	3	5	2	4	3	4
**Asia**	Myanmar	WHO MMR-PDR-2016	2016	32	295	6	12	6	7	0	5
**Asia**	Vietnam	Pham 2015	2009.5	27	463	1	15	1	8	1	5
**Eastern Africa**	Kenya	Frenkel 2017	2014	33	484	3	52	2	44		7
**Eastern Africa**	Kenya	Hamers Ken-Pms-2007-Crh 2011	2007	9	201	2	7	1	4	2	3
**Eastern Africa**	Kenya	Hamers Ken-Pms-2008-Mat 2011	2008	6	200	0	9	0	7	0	1
**Eastern Africa**	Uganda	Hamers Uga-Pms-2008-Jcr 2011	2008	15	179	4	18	1	8	1	10
**Eastern Africa**	Uganda	Hamers Uga-Pms-2008-Jfp 2011	2008	17	185	4	19	2	10	1	11
**Eastern Africa**	Uganda	Hamers Uga-Pms-2008-Mba 2011	2008	2	208	1	23	1	20	0	19
**Eastern Africa**	Uganda	WHO UGA-PDR-2016	2016	9	333	2	44	2	39	0	11
**Latin America**	Argentina	Bissio Arg-Pdr-2014 2017	2014	54	240	12	29	9	21	3	9
**Latin America**	Brazil	De Lourdes Teixeira 2015	2011	56	173	19	17	14	15	9	2
**Latin America**	Guatemala	WHO GTM-PDR-2015	2015	7	234	3	24	2	21	1	7
**Latin America**	Nicaragua	WHO NIC-PDR-2016	2016	21	150	17	23	15	16	8	11
**Southern Africa**	Namibia	WHO NAM-PDR-2015	2015	69	314	20	31	19	27	5	2
**Southern Africa**	South Africa	Gillian Hunt	2016	69	234	29	28	29	26	4	4
**Southern Africa**	South Africa	Hamers Zaf-Pms-2007-Acc 2011	2007	4	207	4	8	4	7	1	1
**Southern Africa**	South Africa	Hamers Zaf-Pms-2007-Mmh 2011	2007	19	177	6	2	4	2	3	0
**Southern Africa**	South Africa	Hamers Zaf-Pms-2007-Tlc 2011	2007	10	173	3	7	2	5	1	2
**Southern Africa**	Zambia	Hamers Zmb-Pms-2007-Chc 2011	2007	4	215	1	11	1	7	1	0
**Southern Africa**	Zambia	Hamers Zmb-Pms-2007-Kar 2011	2007	10	198	4	9	4	7	0	1
**Southern Africa**	Zambia	Hamers Zmb-Pms-2007-Lth 2011	2007	4	100	0	6	0	4	0	3
**Southern Africa**	Zimbabwe	Hamers Zwe-Pms-2008-Con 2011	2008	14	192	3	5	3	3	1	1
**Western/Central**	Cameroon	Billong Cmr-Pms-2009-Yao 2013	2009	2	135	0	4	0	3	0	1
**Western/Central**	Cameroon	Cameroon Who Survey	2015	29	223			3	12	8	2
**Western/Central**	Cameroon	Nanfack Resistance Workshop 2016	2013	48	18				1	0	0
**Western/Central**	Nigeria	Hamers Nga-Pms-2008-Lut 2011	2008	2	193	1	4	1	3	1	1

NRTI = nucleoside reverse transcripate inhibitor ; NNRTI = non-nucleoside reverse transcripate inhibitor ; SDRM = surveillance drug resistance mutation

Supplementary methods

To assess associations between study-level characteristics (e.g. calendar year at midpoint of resistance testing) and drug resistance we performed univariate meta-regression analyses within each region. The meta-regression models were logistic regression models with a random effect at the study level and were done separately within each region (i.e. the same methodology used to pool prevalence within region). The Prior exposure to ARVs was included as the explanatory variable, and drug resistance as the outcome variable.

The output from these meta-regression models were used in three ways. Firstly, by using likelihood ratio tests comparing model fit with and without the inclusion of the exposure variable, we assessed if there was a significant association between exposure and drug resistance. Secondly, by calculating odds ratios, we quantified the estimated extent of any association. Since levels of resistance were generally modest, these odds ratios can be roughly translated into percentage changes on the relative scale. Thirdly, by using the coefficients from these logistic regression models we calculated trend lines showing predicted resistance at any given level of the exposure. We plotted these trend lines against study-specific levels of exposure and drug resistance to present graphically the relationship between exposure and drug resistance amongst the studies we have sampled. For these meta-regression analyses, we generally included all studies with information on the exposure variable. An exception was for prior ARV drug exposure where the analysis was restricted to only studies reporting data in both populations with and without prior drug exposure.

To assess the prevalence of drug resistance within a specified region at any specific time point, we pooled prevalence estimates within the region using an empty logistic regression model with a random effect at the study level. In analyses using calendar time, there was some evidence to suggest that the yearly odds of increase in drug resistance may be constant over time. To formally assess whether odds changed over time, we compared a model with a single yearly odds ratio to a model which allowed a separate odds ratio before and after the midpoint year of sampling (which was 2007). When the model allowing the odds ratio to vary was a significantly better fit to the data (as assessed by comparing the models using a likelihood ratio test with a critical threshold for the p-value of 0.05), we reported odds ratios separately before and after 2007.

This method has previously been shown to perform robustly for meta-analyses of moderately sparse binary data[2]. In studies with no mutations, the proportion of resistance was estimated as 1/4n, where

n was the total number of successful genotypes. Occasionally, for analyses where data were available from only one or a few studies, these logistic regression models did not converge. In these instances-which are highlighted in the tables-we pooled studies using random-effects meta-analysis with DerSimonian-Laird weighting after performing the Freeman-Tukey type arcsine square root transformation $y = \arcsin[\sqrt{(r / (n + 1)}] + \arcsin[\sqrt{(r + 1) / (n + 1)}]$, with a variance of $1 / (n + 1)$. The transformed pooled proportions and 95% confidence intervals were then back-transformed. Statistical analysis was performed in Stata version 14.1 (StataCorp, USA). Meta-analyses with the Freeman-Tukey type arcsine square root transformation were also done to describe the degree of heterogeneity, as measured by the I^2 statistic.[3]

References

1. Hamers RL, Schuurman R, Sigaloff KC, et al. Effect of pretreatment HIV-1 drug resistance on immunological, virological, and drug-resistance outcomes of first-line antiretroviral treatment in sub-Saharan Africa: a multicentre cohort study. *Lancet Infect Dis* 2012; **12**(4): 307-17.
2. Stijnen T, Hamza TH, Ozdemir P. Random effects meta-analysis of event outcome in the framework of the generalized linear mixed model with applications in sparse data. *Stat Med* 2010; **29**(29): 3046-67.

3. Higgins JPT, Thompson SG. Quantifying heterogeneity in a meta-analysis. Stat Med [Internet]. 2002 Jun;21(11):1539–58. Available from: http://dx.doi.org/10.1002/sim.1186
